# School-Related and Individual Predictors of Subjective Well-Being and Academic Achievement

**DOI:** 10.3389/fpsyg.2018.02631

**Published:** 2018-12-21

**Authors:** Ricarda Steinmayr, Anke Heyder, Christian Naumburg, Josi Michels, Linda Wirthwein

**Affiliations:** Department of Psychology, Technical University Dortmund, Dortmund, Germany

**Keywords:** subjective well-being (SWB), academic achievement, school climate, self-efficacy, interest, test anxiety

## Abstract

Recent research in the educational context has focused not only on academic achievement but also on subjective well-being (SWB) as both play a major role in students’ lives. Whereas the determinants of academic achievement have been extensively investigated, little research has been conducted on school-related determinants of SWB in comparison with other students’ characteristics. In the present cross-sectional study, we set out to investigate whether perceived school climate predicts school grades and SWB above and beyond other variables that are important for SWB and academic achievement. A sample of 767 8th and 9th grade students (*n* = 361 female adolescents; age: *M* = 14.07 years, *SD* = 0.92) completed measures of SWB, perceived school climate, test anxiety, self-efficacy, and interest. Grade point average (GPA) indicated students’ academic achievement. Data were analyzed with latent structural equation models in which GPA and SWB were regressed on the school climate variables and students’ characteristics. Results indicated that a positive school climate as well as self-efficacy and the worry component of test anxiety predicted SWB and/or GPA after all other variables were controlled for. Directions for future research and the importance of school climate variables on adolescents’ SWB and academic achievement are discussed.

## Introduction

Positive psychology seeks to shed light on the conditions and processes that contribute to the optimal functioning of human-beings and organizations (Gable and Haidt, 2005). A central construct examined in the context of positive psychology is that of subjective well-being. According to Seligman (2011) subjective well-being is a multidimensional construct and consists of positive emotions, engagement, positive relationships, meaning, and accomplishments or achievements. For adolescents, school is an important source of subjective well-being and recently more emphasis is placed on the fact that schoolchildren should feel comfortable in order to achieve optimal learning conditions (Organisation for Economic Cooperation Development [OECD], 2017). However, it is not only important to find out to what extent an optimal performance is achieved through a climate of well-being but also to explore which individual factors might contribute to a high subjective well-being (Lewis et al., 2011). Researchers as well as practitioners have long acknowledged that both subjective well-being (SWB) and academic achievement are favorable outcomes for students. Concerning the determinants of these outcomes, mostly student characteristics (i.e., individual determinants) have been investigated so far. However, creating a school climate that enables learning and well-being has also been considered important for healthy academic and personal development (e.g., Cohen, 2006). Even though there are strong theoretical assumptions of a link between school climate and these aspects, empirical evidence supporting this association has been scarce. Research on the association between school climate and general SWB has been particularly difficult to find. Thus, so far, little to nothing is known about whether school-related determinants (e.g., school climate) contribute to the prediction of SWB and academic achievement beyond and independently from student characteristics. The aim of the present study is to investigate whether school climate predicts SWB and academic achievement both (a) when important student characteristics that are also known to be important for SWB and academic achievement are controlled for and (b) when they are not.

### On the Role of School Climate

There is a huge number of different conceptualizations of school climate (Wang and Degol, 2016). Many authors agree that school climate is a multidimensional construct. According to the National School Climate Council (2007), “school climate is based on patterns of people’s experiences of school life and reflects norms, goals, values, interpersonal relationships, teaching and learning practices, and organizational structures” (p. 4), most of them including interpersonal variables (e.g., student-teacher relationships) as well as other characteristics (e.g., the atmosphere of a school; see Cohen et al., 2009). In this context, it is important to differentiate between the classroom climate (i.e., the climate surrounding a homogeneous group of students) and the school climate in general (Eder, 2018). The classroom climate, on the one hand, refers to aspects of teaching, how involved the students are, peer relationships, and peer-teacher relationships in rather small groups of students (i.e., a specific class). The school climate, on the other hand, consists of more global dimensions that focus on the school in general (Cohen et al., 2009). In an extensive literature review, Wang and Degol (2016) distinguished between four school climate domains: Academic (quality of the academic atmosphere), community (quality of interpersonal relationships), safety (emotional security, e.g., disciplinary practices), and institutional environment (organizational features of the school environment).

Practitioners and researchers have recently developed great interest in school climate and have acknowledged its importance for students’ learning and healthy development (Eccles et al., 1993; Thapa et al., 2013). In what follows, we summarize empirical evidence on the role of school climate for SWB and academic achievement.

#### School Climate and SWB

Different theoretical approaches to SWB exist and can be differentiated into a hedonic and an eudaimonic view of SWB (e.g., Ryan and Deci, 2001; Eid and Larsen, 2008). The hedonic view defines SWB as the presence of joy or happiness. In this context, most authors differentiate between a cognitive and an affective component of SWB (see Diener et al., 1999; Diener, 2012). The cognitive component describes individuals’ cognitive evaluations of their lives as a whole (i.e., global life satisfaction). The affective component comprises affective experiences, including individuals’ reports of pleasant emotions (e.g., joy, enthusiasm) and negative emotions (e.g., sadness, nervousness; e.g., Watson et al., 1988). The eudaimonic view focuses on self-realization and defines well-being as the degree to which an individual is fully functioning. As there are different conceptualizations regarding eudaimonic well-being and empirical uncertainties concerning the factorial structure of eudaimonic well-being measures (Springer and Hauser, 2006), we refer to SWB as defined in the hedonic approach. Hence, we focus on SWB conceptualized as cognitive as well as affective components. There are already some studies investigating individual determinants of school students’ SWB such as different personality variables (e.g., Anglim and Grant, 2014). However, research focusing on different school variables such as school climate as determinants of adolescents’ SWB is still scarce. In the following, we refer to different theoretical approaches linking school climate and SWB.

Ecological system theories assume an impact of family, school, and other layers of the environment on children’s and adolescents’ positive development (e.g., Bronfenbrenner, 1979). In this context, schools might have an important influence as an environment that contributes to a healthy and positive adjustment and hence, to the well-being of children and adolescents (Baker et al., 2003). Baker et al. (2003) refer to different aspects of school climate as distal environmental aspects that influence well-being.

The stage-environment fit approach by Eccles et al. (1993) is grounded in the person-environment fit paradigm and is consistent with this theory. According to this approach, children’s healthy development is possible only if the environment fulfills the prerequisites for a healthy development. On the basis of these theoretical approaches, one might assume that SWB, as one sign that a student is developing in a healthy way, is impacted by a positive school climate because schools constitute a very important environment for children and adolescents given the amount of time they spend in school.

Indeed, research investigating the association between a positive school climate and students’ SWB is scarce. Only a few studies have investigated the relationships between the two components of SWB (i.e., cognitive and affective) and school climate and found them to be positively correlated (e.g., Waaler et al., 2013; Newland et al., 2014). However, the school climate measures used by these authors were either very short (Newland et al., 2014) or they did not directly measure school climate but instead measured constructs associated with it in a broader sense (Waaler et al., 2013). Most studies using established measures of school climate have focused on either life satisfaction or symptoms associated with the affective component of SWB (e.g., depression and anxiety symptoms). To our knowledge, only one study investigated school climate (e.g., teacher support, school connectedness) with an established measure of the affective component of SWB and found significant and direct, albeit small effects of school climate variables on affective SWB (Aldridge et al., 2016). Concerning life satisfaction, Suldo and Huebner (2006) demonstrated that students with very high life satisfaction perceived the greatest social support from teachers, which is one component of school climate. Furthermore, high-school students’ life satisfaction was positively associated with order and discipline, the sharing of resources, parental involvement, the appearance of school buildings, students’ interpersonal relationships, and student-teacher relationships (Suldo et al., 2006, 2008).

Also, findings from studies that did not explicitly measure SWB have supported the link between school climate and the affective component of SWB. These studies differed regarding the assessment of school climate and used either a global school climate score or scores from specific subfacets of school climate. Lee et al. (2017) focused on depression when investigating the criterion validity of the School Climate and School Identification Measure-Student measure (SCSIM-St). The total score, comprising student-student relationships, student-staff relationships, academic emphasis, and shared values was significantly negatively correlated with depression in an adolescent student sample. However, the authors considered only the total score and did not report the correlations between the subscales of the SCSIM-St and depression. Similar results were found when teacher-reported school climate was assessed, that is, teacher-reported school climate was also negatively associated with students’ depression scores (Pössel et al., 2016). Salmela-Aro et al. (2008) considered three subscales of school climate and regressed them on school burnout, which might also be interpreted as an indicator of the affective component of SWB. All school climate factors were correlated with school burnout (positive correlation: negative school climate; negative correlation: support from school, positive motivation from teachers). Correlations between the school climate factors were high: *r* > | 0.5|. However, when all three school climate factors were simultaneously regressed on school burnout, all of them significantly contributed to the prediction of school burnout on an individual level but not on the school level. On each level, the path weights for the different school climate factors differed from each other, as well. Thus, whereas most studies have explored a global school climate factor, considering specific factors of school climate may provide a better understanding of how school climate contributes to the explanation of individual differences in SWB or related aspects. The global school climate factor might mask effects of single school climate factors. In summary, studies have supported a positive association between positive components of school climate and SWB or constructs associated with SWB such as depression. These correlations have usually been medium to large in size.

#### School Climate and Academic Achievement

School climate and academic achievement are thought to be positively associated because a good school climate is a prerequisite for learning (Thapa et al., 2013). Likewise, Osher and Kendziora (2010) stated that a negative school climate may limit students’ school engagement, which might subsequently lead to worse academic achievement. In line with these thoughts, studies have demonstrated a positive relation between school climate variables such as student-teacher relationships and prerequisites for learning such as students’ academic motivation, school engagement, or attitudes towards school (e.g., Fraser and Fisher, 1982; Wang and Holcombe, 2010; Van Ryzin, 2011). Moreover, academic self-efficacy has also been found to be positively associated with a good school climate as assessed by feelings of connectedness with students’ schools (McMahon et al., 2009) or perceived support from teachers (Alivernini and Lucidi, 2011). But school climate is associated not only with prerequisites for academic achievement but also with academic achievement itself. Several studies have demonstrated a positive association between school climate and different indicators of academic achievement (GPA: e.g., Suldo et al., 2008; standardized school achievement: e.g., Lee et al., 2017). An association with academic achievement was found at both the individual and school levels (e.g., Salmela-Aro et al., 2008), but the associations were small in size (*r*< |0.3|). Furthermore, students who were held back a grade also felt less connected to school (e.g., Fan et al., 2011). So there is some evidence that a positive school climate is associated with important prerequisites for academic achievement and academic achievement itself, or in other words, that a negative school climate might have a negative impact on academic achievement and its prerequisites. However, it also makes sense to expect that academic difficulties might lead to a negative perception of school climate. Longitudinal studies are needed to investigate this relation. We are aware of only one study that investigated the reciprocal effect between academic achievement and school climate by assessing both constructs over time. McCoy et al. (2013) found that school climate predicted change in academic achievement but not vice versa on a school level. Even though school level results are not necessarily applicable to the individual level, we propose that school climate is a predictor of academic achievement rather than vice versa on the basis of Osher and Kendziora (2010) and Thapa et al. (2013) rationales and McCoy et al.’s (2013) study.

### On the Role of Student Characteristics

Besides school climate, student characteristics are also important for students’ SWB and students’ academic achievement. In the following paragraphs, we summarize the empirical findings on the student characteristics that predict SWB and achievement. We thereby focus on three constructs that are assumed to be relevant for both outcome variables (i.e., self-efficacy, interest, and test anxiety). In motivation research, there is a problem with jingle-jangle fallacies as theoretically very similar constructs are given different names (Marsh et al., 2003). Marsh et al. (2003) proposed that motivational constructs can be attributed to either a learning factor (e.g., intrinsic motivation or related constructs) or to a performance factor (e.g., achievement motivation). By choosing interest and self-efficacy, we considered both motivation factors in the present study. Self-efficacy is defined as “beliefs in one’s capabilities to organize and execute the courses of action required to produce given attainments” (Bandura, 1997, p. 3). Individual interest can be defined as a relatively enduring preference for a certain type of object, activity, or subject (e.g., Schiefele, 1991).

Furthermore, personality variables have been found to be the most important individual characteristics for SWB (e.g., Anglim and Grant, 2014). However, we did not include broader personality factors such as the Big Five in our study because only conscientiousness has been found to be related to GPA in adolescence (Poropat, 2009), and conscientiousness is not related to SWB (Gomez et al., 2009). On the other hand, neuroticism is related to SWB (Gomez et al., 2009) but not to GPA (Poropat, 2009). An important construct in the context of school that shows substantial associations with personality variables is test anxiety. It is an important indicator of neuroticism, it has the highest association with SWB of all personality variables (e.g., Weiss et al., 2008), and it has substantial associations with academic achievement (e.g., Hembree, 1988; Zeidner, 1998; Steinmayr et al., 2016). Test anxiety refers to a set of different “phenomenological, physiological, and behavioral responses that accompany concern about possible negative consequences” in an evaluative situation (Zeidner, 2007, p. 166). It can be conceptualized as a state or trait, whereby a frequently used definition refers to test anxiety as a situation-specific personality trait (Spielberger and Vagg, 1995). Test anxiety consists of two components: emotionality and worry (e.g., Hembree, 1988; Cassady and Johnson, 2002). Emotionality comprises a person’s physiological state such as nervousness, an accelerated heart rate, or tension when confronted with tests. The worry component is comprised of cognitive elements of test anxiety such as negative thoughts, self-criticism, or concerns about the effects of failure (Zeidner, 1998).

#### Student Characteristics Predicting SWB

Research has shown that individual variables are strong predictors of SWB (Diener, 2012). However, individual variables focusing on the academic or school context have still rarely been examined so far (Huebner and Diener, 2008). This is surprising given the fact that children and adolescents spend most of their time in school, and hence, school-related individual determinants would have to play an important role in predicting SWB. Concerning self-efficacy, Correia and Dalbert (2007) found a correlation of *r* = 0.49 between school-related self-efficacy and life satisfaction in a sample of school students. Drawing on a sample of undergraduate students, academic self-efficacy was positively related to life satisfaction (*r* = 0.40) as well as to positive affect (*r* = 0.39) and negative affect (*r* = -0.26; Denovan and Macaskill, 2017).

Further, several studies investigated the relations between SWB in school or university and interest or related constructs such as intrinsic motivation and revealed positive relations (e.g., Baker, 2004; Ruus et al., 2007). Given that Suldo et al. (2008) demonstrated a medium to high correlation between school satisfaction and general life satisfaction, we expected that interest would also be associated with general SWB. However, even less is known about other school-related determinants of SWB besides motivational variables.

Regarding the influence of test anxiety on SWB, the transactional model of test-related emotions (Spielberger and Vagg, 1995; Zeidner, 1998; Ringeisen and Buchwald, 2010) suggests that test anxiety is associated not only with appraisals of threat but also with other negative emotions, and hence, test anxiety may predict changes in SWB. Steinmayr et al. (2016) focused on the associations between two components of test anxiety (worry, emotionality) and SWB. The authors found that worry negatively predicted changes in life satisfaction and changes in affective well-being. Studies focusing on variables considered to also indicate emotional well-being have additionally shown associations with test anxiety (e.g., Pekrun et al., 2002). Taken together, various studies have suggested that self-efficacy and interest are positive predictors of SWB, whereas test anxiety, and worry in particular, is a negative predictor.

#### Student Characteristics Predicting Academic Achievement

Much research has studied the role of student characteristics such as students’ motivation and emotions in students’ achievement (e.g., Eccles and Wigfield, 2002; Gogol et al., 2017; Pekrun et al., 2017). Students’ self-efficacy has been found to influence students’ choice of activities, effort, persistence, and eventually achievement (e.g., Zimmerman, 2000). This means that regardless of their prior achievement, students who judge their own capability for learning and achievement as high choose more challenging tasks, put more effort into them, show higher persistence, and thus show higher performance than students who judge their self-efficacy as low (for a summary, see Zimmerman, 2000). Empirical research has underscored the power of self-efficacy beliefs, which explain around 25% of the variance in academic outcomes (Pajares, 2006). Prior research has suggested that self-efficacy is an even stronger predictor of achievement than other motivational constructs such as self-concept or utility value (Pajares and Miller, 1994; but see Steinmayr et al., 2018). Note, however, that due to the very strong correlations between self-efficacy and self-concept, researchers should be cautious when making such interpretations (see Marsh et al., 2004).

Research has also demonstrated that interest increases attention, recall, task persistence, and effort (e.g., Ainley et al., 2002; Hidi and Renninger, 2006). A meta-analysis on the relation between interest and performance revealed moderate, positive correlations between the two constructs (Schiefele et al., 1992).

Regarding the role of students’ emotions for learning and achievement, most research has focused on test anxiety (e.g., Hembree, 1988; Zeidner, 1998). Generally, negative activating emotions such as anxiety distract attention and reduce interest, intrinsic motivation, and deep learning, but sometimes they can also increase students’ extrinsic motivation in an attempt to avoid failure (Pekrun, 2017). In cross-sectional studies, negative small to moderate correlations have been found between test anxiety and achievement with stronger relations for the worry component than the emotionality component (e.g., Hembree, 1988). In longitudinal research, results on the effects of test anxiety on achievement outcomes have been less conclusive. Older studies revealed an indirect negative effect of test anxiety on achievement via students’ motivation but no direct effect (Meece et al., 1990). More recently, worry but not emotionality was found to predict a decrease in students’ GPA (Steinmayr et al., 2016), reflecting the correlational pattern established in prior research (Hembree, 1988).

Taken together, self-efficacy, individual interest, and test anxiety are well-researched motivational-affective key constructs in educational psychology research. Self-efficacy and interest positively predict achievement, whereas test anxiety—particularly the worry component—seems to decrease academic achievement.

### Academic Achievement and SWB

Prior research has suggested that school climate, students’ self-efficacy, interest, and test anxiety show significant associations with SWB and with achievement in the same direction. So how are these two student outcomes related? Several studies have already explored the link between SWB and academic achievement. Life satisfaction, in particular, seems to be positively related to adolescents’ academic achievement (e.g., Gilman and Huebner, 2006; Proctor et al., 2010; Heffner and Antaramian, 2016). More specifically, the correlations between life satisfaction and grade point average (GPA) were found to range from *r* = 0.12 (Verkuyten and Thijs, 2002) to *r* = 0.32 (Gilman and Huebner, 2006). Regarding affective measures of SWB, the associations with GPA have been more heterogeneous and usually smaller. Whereas Heffner and Antaramian (2016) found significant, albeit small correlations (*r* = -0.15 with negative affect and *r* = 0.07 with positive affect) in a sample of school students, no significant correlation between positive affect and GPA was found in studies with university students (Liao and Wei, 2014). Taken together, life satisfaction has been found to be positively related to grades in school, and the association tends to be higher than for the affective component of SWB.

### The Present Study

As outlined, the association between school climate and SWB has seldom been investigated with established measures of the two components of SWB (i.e., affective and cognitive) and school climate. However, research on the relations between school climate and constructs related to SWB has supported the notion that school climate and SWB are related. However, most studies have investigated either one aspect of school climate (e.g., Shochet et al., 2006; Suldo and Huebner, 2006) or a global school climate score (Lee et al., 2017). Little is known about the importance of specific aspects of school climate for the different components of SWB (but see Aldridge et al., 2016). Salmela-Aro et al. (2008) demonstrated that the school climate subscales differ in their criterion validity concerning SWB when considered simultaneously. However, they did not test whether the highly correlated scales would also have incremental validity after a general school climate factor was controlled for. Thus, one aim of the present study is to investigate whether the school climate subscales differ in their associations with SWB and whether they predict SWB above and beyond a general school climate factor. Because very few studies have investigated school climate and SWB, and most studies have considered either a global measure of school climate or its subscales, we have no specific hypotheses regarding which subscale should best predict SWB or whether the subscales should predict SWB beyond a general school climate factor.

Research Question 1a: Can the school climate subscales predict SWB beyond a general school climate factor?
Research Question 1b: Can the school climate subscales predict academic achievement beyond a general school climate factor?

As described above, school climate is an important predictor not only of SWB but also of academic achievement. However, only a few studies have investigated both criteria simultaneously with regard to their associations with school climate. In one study, school climate had a stronger association with SWB than with academic achievement when bivariate correlations were considered (see Salmela-Aro et al., 2008). This result is supported by comparisons of different studies that have investigated (aspects of) either SWB or academic achievement. Theories explaining SWB or students’ healthy development (e.g., Bronfenbrenner, 1979; Eccles et al., 1993) have put more focus on the environment than theories explaining academic achievement (for an overview, see Steinmayr et al., 2014). In these theories, a positive environment is one prerequisite for academic achievement among many others. On the basis of these theoretical considerations and the empirical support for them, we expected that school climate would be more strongly associated with SWB than with academic achievement.

Hypothesis 1: School climate will be a stronger predictor of SWB than of academic achievement.

This finding should also hold true when further determinants of SWB and academic achievement are considered. Here, we concentrated on how students’ characteristics should be important for both SWB and academic achievement. We are not aware of a study that has investigated the incremental validity of school climate variables beyond individual students’ characteristics such as self-efficacy, text anxiety, and interest, when explaining individual differences in the two components of SWB (i.e., affective and cognitive) and academic achievement. However, due to the importance of school climate for students’ SWB, we would expect school climate to predict SWB above and beyond other important individual student characteristics such as self-efficacy, interest, and test anxiety. This view is supported by the study by Suldo et al. (2008) who found that school climate predicted global life satisfaction beyond personal academic beliefs and other predictors of SWB. However, we predict that school climate will be less important for academic achievement when simultaneously considered with individual student characteristics that are important for school success. In their opportunity-propensity framework for explaining academic achievement, Byrnes and Miller (2007) included school climate as an opportunity factor, whereas student characteristics such as motivation were considered as propensity factors. When regressed together on academic achievement, the opportunity factors were frequently demonstrated to be less important for academic achievement than the propensity factors (e.g., Byrnes and Miller, 2007; Lewis and Farkas, 2017). In line with these thoughts is the result that school climate does not predict academic achievement above and beyond other variables, such as self-efficacy (Alivernini and Lucidi, 2011; Wang et al., 2013). On the basis of these findings, we derived the following hypothesis:

Hypothesis 2: School climate will predict SWB above and beyond self-efficacy, interest, and test-anxiety but not academic achievement.

## Materials and Methods

### Sample and Procedure

The sample comprised 775 students from four different schools (two comprehensive schools and two schools of the school type “Gymnasium,” the school type attended by academic track students) in Germany that were contacted by research assistants. Eight students were excluded from the analyses because they either did not answer most parts of the questionnaires or just made patterns with their answers. We further checked for outliers via regression analysis but no exclusion was necessary. Thus, the final sample consisted of 767 students (361 female adolescents). Of those, 390 students attended a comprehensive school and 377 a Gymnasium. At each school, the entire 8th and 9th grades participated (i.e., 33 classes in total). Students were on average 14.07 (*SD* = 0.82) years old, and 152 students reported an immigration background (i.e., they were not born in Germany, did not learn German as a first language, or spoke a language other than German at home). Most students who had an immigration background were associated with Turkey. Students indicated that *n* = 293 fathers and *n* = 232 mothers had a school leaving certificate that qualified them for university. However, *n* = 186 gave no information about the highest school leaving certificate of their fathers, and *n* = 174 gave no information about the highest school leaving certificate of their mothers. Thus, parents’ education was on average higher and immigration background lower than would be the case in a representative student sample, a finding that can be explained by the high percentage of students attending a Gymnasium in the present sample (cf. Steinmayr et al., 2018). We checked for outliers but no exclusion was necessary.

All achievement criteria and all predictors were assessed at the end of 2015 or at the beginning of 2016. Participation in the study was voluntary, and students were allowed to participate only if they provided an informed consent form from one of their parents. More than 95% of all parents agreed that their child was allowed to participate. In addition to parent refusals, another 10% of the overall student population did not participate due to illness, extra-curricular activities, or other reasons unrelated to the testing. Questionnaire administration took place during a regular class in schools. The measures were administered by trained research assistants and lasted about 95 min.

### Measures

#### School Climate

*School climate* was measured using the German *Linz Questionnaire of School and Class Climate for grades 8-13* (LFSK, Eder, 1998). The school climate questionnaire consists of 27 items. These items can either be summed into a total score indicating school climate in general or into four subscales (*discipline/control*, *stimulation/activities*, *warmth*, and *performance orientation*) indicating correlated but specific aspects of school climate. Students were asked to indicate on a 5-point Likert rating scale ranging from 1 (*totally disagree*) to 5 (*totally agree*) how the items applied to them. The *discipline/control* scale assesses the extent to which rules are clearly defined at school and the extent to which they are enforced (e.g., “At this school, there are clear rules for how students should behave”; six items). The second scale *stimulation/activities* assesses the extent to which the school provides extracurricular activities for its students.(e.g., “There are many opportunities for students to pursue their hobbies at this school”; five items). The third scale w*armth* measures the extent to which students rate their school, especially their teachers, as supportive and caring. It comprises the quality of student-teacher relationships (e.g., “In general, our teachers are supportive,” six items) and the atmosphere at the school (e.g., “Mostly the atmosphere at our school is friendly”; three items). The fourth scale *performance orientation* assesses the level of performance expectations in the school (e.g., “Students are expected to work hard and perform well”; seven items). The internal consistency of all subscales was at least satisfactory with the exception of *discipline/control* (*discipline/control*: α = 0.62; *stimulation/activities*: α = 0.72; w*armth:* α = 0.87; *performance orientation:* α = 0.76). The internal consistency of the total school climate score was high (α = 0.83).

#### Self-Efficacy

Self-efficacy was measured with the German translation of the *self-efficacy* subscale taken from the *self-regulation questionnaire* by O’Neil and Herl (1998). This scale was already successfully applied in PISA 2000 in Germany (Kunter et al., 2002, p. 166). It consists of four items that are rated on a 5-point Likert rating scale ranging from 1 (*totally disagree*) to 5 (*totally agree*). An example item is “I am certain I can understand the most difficult material presented in the reading for school.” The internal consistency of the scale was high (α = 0.80).

#### Interest

To assess students’ interest in school in general, we adapted the three German items included in PISA 2000 to measure students’ interest in math (Kunter et al., 2002, p. 166). This means that we replaced “math” with “school” in the wording of the items (e.g., “When I work on my school work, I sometimes get totally absorbed”). Each item was rated on a scale ranging from 1 (*totally disagree*) to 5 (*totally agree*). Cronbach’s alpha was satisfactory (α = 0.74).

#### Test Anxiety

This construct was assessed with a short version (Schwarzer and Jerusalem, 1999) of the *German Test Anxiety Inventory* (TAI-G; Hodapp, 1991, 1995), a revised multidimensional version of *Spielberger’s Test Anxiety Inventory* (TAI; Spielberger et al., 1981). The short version consists of the scales *worry*, which assesses how much a person worries in test situations (five items), and *emotionality*, which assesses the physiological and excitement-related components of a test situation (five items). The students were instructed to use a 4-point Likert rating scale ranging from 1 (*almost never*) to 4 (*almost always*) to rate how they typically feel (e.g., “My heart is in my mouth”) and what they usually think (e.g., “I ask myself whether my performance will be sufficient”) in test situations. The internal consistencies of both scales were good (emotionality: α = 0.78; worry: α = 0.84).

#### Academic Achievement

Academic achievement was measured with grade point average (GPA) as indicated by students’ self-reports. In Germany, grades are coded so that 1 indicates outstanding achievement and 6 indicates the poorest achievement. Grades were reversed to facilitate interpretation of the results so that higher scores indicated better performance.

#### Subjective Well-Being (SWB)

Subjective well-being was measured with a short version of the German Habitual SWB Scale (HSWBS; Dalbert, 2003). The original scale consists of a mood-level scale (Dalbert, 1992) (six items) and a satisfaction with life scale (Dalbert et al., 1984) (seven items). The mood level scale assesses the affective (i.e., emotional) component of SWB, whereas the satisfaction with life scale assesses the cognitive component of SWB. Due to the time restrictions that are common in school testing, we used a short version of each scale, which comprised five items for each subscale (mood level scale: e.g., “Mostly I am happy”; life satisfaction: e.g., “I am content with my life”).

### Statistical Analyses

Data were nested in schools but only partly in classes. Students attending a Gymnasium were organized in classes. However, students attending comprehensive school were still organized in classes but main subjects (e.g., English, mathematics, and German) were taught at different achievement levels (basic and advanced) realizing arrangements for differentiated education which involves tailoring the curriculum to different ability groups of students within the same school (Mitter and Shaw, 1991). Thus, comprehensive school students attending the same class attended different courses in each of these subjects. As a consequence, an indicator based on grades in different subjects as the GPA was not nested into classes for the Comprehensive school students. Thus, the class level was not considered in the analysis as not all data were nested in classes. However, even though all data were nested in schools we did not apply any features of multilevel modeling, as four schools were not enough clusters on the second level (Nezlek, 2011). To account for variance due to different schools, all data were z-standardized within schools before the analyses were run. Data were analyzed with latent Structural Equation Modeling (SEM) computed with Amos 25.0. For the evaluation of overall model fit, three different fit indices were used (see Hu and Bentler, 1999): χ^2^ value, Root Mean Square Error of Approximation (RMSEA), and Comparative Fit Index (CFI). Hu and Bentler (1999) proposed the following cut-off scores for two of these indices: CFI ≥ 0.95 and RMSEA ≤ 0.05. According to Browne and Cudeck (1993), RMSEA ≤ 0.05 indicates a very good model fit and RMSEA ≤ 0.09 is still an indicator of a reasonable error of approximation. According to Hu and Bentler (1995), it is difficult to provide a recommended range for the CFI because, in some cases, even CFI < 0.90 can indicate a reasonable model fit (see also Heene et al., 2011).

There were only small amounts of missing data for individual items (less than 1%) with the exception of self-reported GPA (5%). We compared data for those students who reported GPA versus those students who did not. The two groups did not show statistically significant differences on any of the variables we investigated. Thus, as proposed by several authors, we accounted for missing data by applying full information maximum likelihood (FIML) estimation (Enders and Bandalos, 2001; Newman, 2003).

**FIGURE 1:**
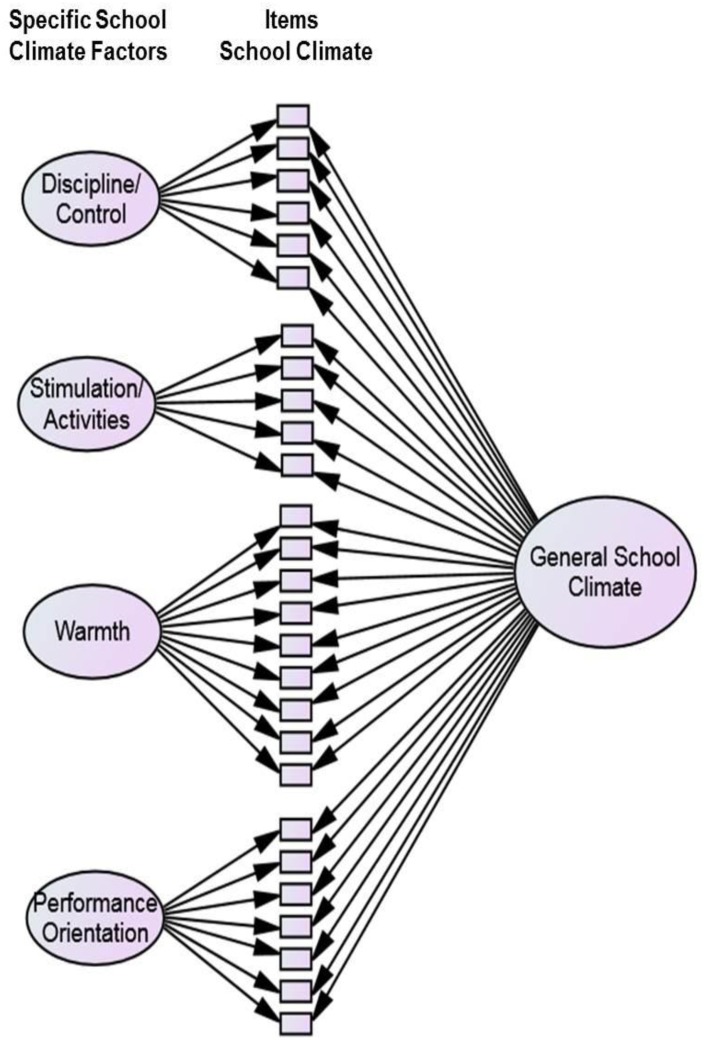
Nested factor model for modeling school climate.

Research Questions 1a and 1b were tested with SEM in which school climate was specified as a nested factor model (e.g., Gustafsson and Balke, 1993; Brunner et al., 2012). As described above, the items can either be summed into a general score or into four subscales (discipline/control, stimulation/activities, warmth, and performance orientation). The nested factor models allowed us to test the relative importance of both the general and specific factors of school climate in one model. Figure 1 illustrates how we modeled school climate in the present study. To answer Research Questions 1a and 1b, either SWB or academic achievement was regressed on the general school climate factor as well as on the other four specific school climate factors depicted in Figure 1.

**FIGURE 2:**
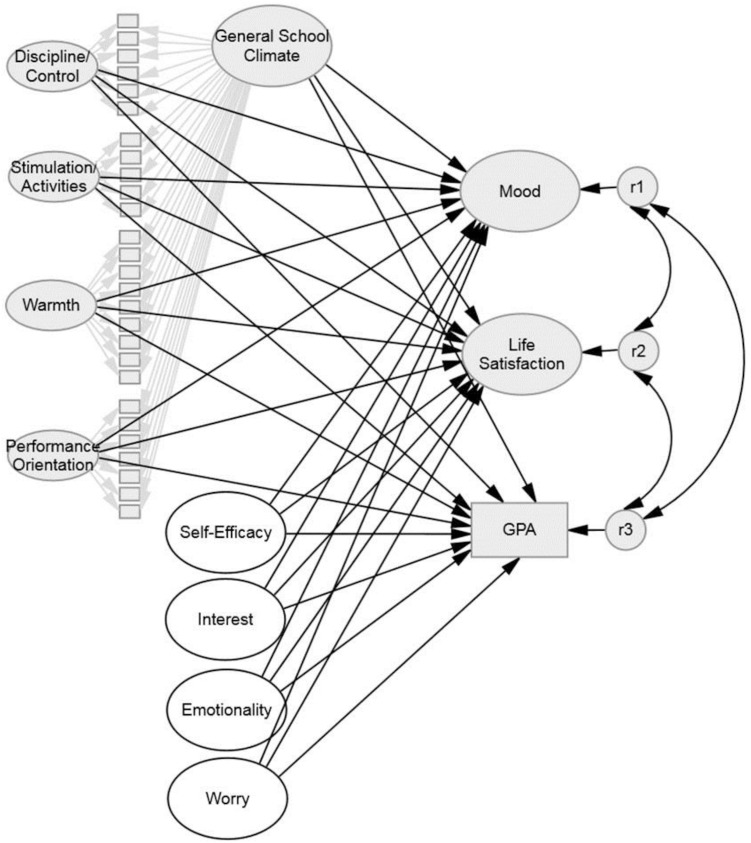
The whole model that was tested. For reasons of clarity, correlations between exogenous factors are not depicted. Results are displayed in Table 3. Constructs displayed with a gray background were tested in Model 1: SWB, Model 2: GPA, and Model 3: SWB + GPA. These results are displayed in Table 2.

To test Hypothesis 1, both SWB and academic achievement were regressed on the general school climate factor and the school climate subscales (see Figure 2, constructs depicted by light gray symbols). Then, the paths from school climate to SWB and academic achievement were set equal to each other. A significantly poorer fit of the constrained model with equated path weights compared with the baseline model in which all paths were freely estimated would indicate that criterion validity differs for the dependent variables. The change in model fit was evaluated by calculating the scaled chi-square difference test (Satorra and Bentler, 2001). In the case of a poorer fit of the constrained model, pairwise comparisons were performed to examine which paths differed from each other. Hypothesis 3 was tested in one model in which SWB and academic achievement were simultaneously regressed on school climate, self-efficacy, interest, and test anxiety. This whole model is presented in Figure 2.

## Results

### Descriptive Statistics

The means, standard deviations, and internal consistencies for all measures as well as their intercorrelations are presented in Table 1. Most correlations were comparable to those found in the literature beside the following correlations: interest did not correlate with mood and emotionality did not correlate with GPA.

**Table 1:** Means (*M*), standard deviations (*SD*), internal consistencies (α), and intercorrelations among all observed predictors and criteria.

	Descriptives			Intercorrelations											
	*M*	*SD*	α	2	3	4	5	6	7	8	9	10	11	12
(1) GPA	2.40	0.65	–	0.23	0.18	0.12	-0.02	0.09	0.21	>0.01	0.30	0.08	-0.05	-0.12
(2) Mood	4.33	0.88	0.74		0.58	0.23	0.00	0.23	0.25	0.07	0.25	0.06	-0.11	-0.16
(3) Life satisfaction	4.86	0.89	0.83			0.25	0.02	0.21	0.27	0.12	0.24	0.08	-0.12	-0.17
(4) General school climate	3.24	0.46	0.83				0.54	0.73	0.73	0.69	0.21	0.19	-0.03	-0.02
(5) Discipline/control	3.26	0.64	0.62					0.23	0.09	0.30	0.00	0.11	0.04	0.11
(6) Stimulation/activities	2.85	0.81	0.72						0.42	0.36	0.16	0.13	-0.02	-0.07
(7) Warmth	3.46	0.65	0.87							0.24	0.24	0.11	-0.11	-0.14
(8) Performance-orientation	3.22	0.64	0.76								0.14	0.19	0.04	0.11
(9) Self-efficacy	3.35	0.73	0.80									0.40	-0.20	-0.14
(10) Interest	2.59	0.88	0.74										-0.09	-0.01
(11) Emotionality	1.94	0.62	0.78											0.55
(12) Worry	2.72	0.80	0.84											-

*N = 767; The SWB scales (life satisfaction and mood) ranged from 1 to 6, the school climate scales ranged from 1 to 5 (Discipline/Control, Stimulation/Activities, Warmth, Performance Orientation), the self-efficacy scale ranged from 1 to 5, the interest scale ranged from 1 to 5, the test anxiety scales (Emotionality and Worry) ranged from 1 to 4 with 1 indicating lower emotionality/worry. GPA: grades were recoded so that higher values indicate higher academic achievement; r ≥∣0.08∣, p < 0.05; r ≥∣0.09∣, p < 0.01*.

### Incremental Validity of Specific School Climate Scales

Research Questions 1a and 1b addressed the incremental validity of specific school climate scales above and beyond the general school climate factor. First, we checked on the model fit of the nested school climate factor model depicted in Figure 1. Model fit was very good, χ^2^(291) = 632.89, *p* < 0.001; RMSEA = 0.039, 90% CI [0.035, 0.043]; CFI = 0.936. Then we set up two models in which either SWB or academic achievement was regressed on school climate modeled as depicted in Figure 1 [Model 1: SWB and Model 2: GPA]. SWB was modeled as two correlated factors (i.e., mood and life satisfaction), each indicated by five manifest variables (i.e., items). Academic achievement was modeled as a manifest variable. For both models, the model fit indices indicated an excellent fit to the data, SWB: χ^2^(585) = 1037.73, *p* < 0.001; RMSEA = 0.032, 90% CI [0.029, 0.035]; CFI = 0.945; Academic Achievement: χ^2^(319) = 766.75, *p* < 0.001; RMSEA = 0.043, 90% CI [0.039, 0.047]; CFI = 0.917. Table 2 displays the path coefficients for the general school climate factors as well as for the specific school climate factors in predicting SWB and academic achievement. Only the general school climate factor was a significant predictor of both SWB and GPA. In both models, the specific school climate factors did not contribute to the prediction.

**Table 2:** Model Fit Indices and unstandardized (b) as well as standardized (β) path weights of the SEM in which SWB and academic achievement were regressed on school climate.

Model	X^2^ble	CFI	RMSEA	Predictor	Mood			Life satisfaction			GPA		
						
					*b*	*SE*	β	*b*	*SE*	β	*b*	*SE*	β
(1) SWB	1.943	0.932	0.035	SC-general	1.127*	0.192	0.424	0.743*	0.137	0.368			
				SC – d/c	-0.128	0.096	-0.061	-0.057	0.073	-0.036			
				SC – s/a	0.179	0.109	0.08	0.076	0.082	0.044			
				SC – w	0.055	0.045	0.073	0.026	0.034	0.046			
				SC – po	-0.06	0.105	-0.026	0.006	0.079	0.003			
(2) GPA	2.404	0.917	0.043	SC-general							0.683*	0.185	0.19
				SC – d/c							-0.077	0.131	-0.028
				SC – s/a							0.043	0.144	0.015
				SC – w							-0.013	0.06	-0.013
				SC – po							-0.07	0.142	-0.024
(3) SWB	1.940	0.929	0.035	SC-general	1.127*	0.192	0.424	0.744*	0.137	0.367	0.667*	0.18	0.19
+ GPA				SC – d/c	-0.128	0.096	-0.061	-0.057	0.073	-0.036	-0.091	0.131	-0.033
				SC – s/a	0.181	0.109	0.081	0.077	0.082	0.045	0.025	0.147	0.009
				SC – w	0.055	0.045	0.073	0.026	0.034	0.046	-0.027	0.06	-0.027
				SC – po	-0.06	0.104	-0.026	0.006	0.079	0.003	-0.072	0.143	-0.024

*SC, school climate; d/c, *discipline/control*; s/a, *stimulation/activities*; w, w*armth* and po, *performance orientation*; GPA, Grade Point Average, grades were recoded so that higher GPA reflects better academic achievement. ^∗^*p* ≤ 0.001*.

### Differential Effects of School Climate

To test Hypothesis 1, we set up a model with three correlated criteria (mood, life satisfaction, and GPA) that were regressed on school climate [Model 3: SWB + GPA; see also Figure 2, light gray]. In order to test Hypothesis 2, the paths from general school climate to the two components of SWB and academic achievement were set equal. Then the constrained model was tested against the baseline model in which these paths were freely estimated. Setting the paths from the general school climate factor to the three criteria to equality led to a significant deterioration in model fit (Δχ^2^ = 212.758, Δ*df* = 2, *p* < 0.001). A subsequent analysis demonstrated that the path coefficient from general school climate to mood was significantly higher than the ones from general school climate to academic achievement (Δχ^2^ = 6.249, Δ*df* = 1, *p* = 0.012) and to life satisfaction (Δχ^2^ = 12.745, Δ*df* = 1, *p* < 0.001). The path coefficients from general school climate to life satisfaction and academic achievement were not significantly different (Δχ^2^ = 0.192, Δ*df* = 1, *p* = 0.661). Thus, Hypothesis 1 was supported for mood but not for life satisfaction.

### Incremental Validity of School Climate and Students’ Characteristics

To test Hypothesis 2, we set up a latent SEM in which SWB and academic achievement were regressed on school climate, self-efficacy, interest, and test anxiety (see Figure 2, the whole model). The residuals of GPA and both SWB components were correlated, and so were all exogenous factors beside the school climate factors which were only correlated with self-efficacy, interest, and test anxiety but not with themselves (cf. Figure 1). Except for GPA, all constructs in the model were latent. The data fit the model very well: χ^2^(1348) = 2399.64, *p* < 0.001; RMSEA = 0.032, 90% CI [0.030, 0.034]; CFI = 0.923. Results are displayed in Table 3.

**Table 3:** Model fit indices and path weights of the SEM in which SWB and academic achievement were regressed on school climate, self-efficacy, interest, and test anxiety.

Predictor	Mood			Life satisfaction			GPA		
Path weight	*b*	*SE*	β	*b*	*SE*	β	*b*	*SE*	β
SC-general	0.973**	0.191	0.35	0.588**	0.132	0.277	0.29	0.18	0.079
SC – d/c	-0.052	0.097	-0.024	0.009	0.074	0.005	0.015	0.13	0.005
SC – s/a	0.175	0.104	0.081	0.066	0.078	0.04	-0.001	0.136	<0.001
SC – w	0.066	0.044	0.087	0.024	0.033	0.041	0.005	0.058	0.005
SC – po	-0.054	0.102	-0.025	0.029	0.077	0.017	-0.15	0.136	-0.052
Self-Efficacy	0.176*	0.068	0.158	0.088	0.051	0.103	0.531**	0.092	0.361
Interest	0.012	0.059	0.013	0.053	0.044	0.072	0.035	0.078	0.028
Emotionality	0.053	0.081	0.045	0.03	0.061	0.033	0.205	0.107	0.131
Worry	-0.139	0.073	-0.123	-0.147*	0.056	-0.171	-0.14	0.097	-0.094

*Model Fit: CMIN = 1.780; CFI = 0.923; RMSEA = 0.032 (90%-CI: 0.030–0.034). Model is depicted in Figure 2. SC, school climate; d/c, discipline/control; s/a, stimulation/activities; w, warmth, and po, performance orientation; GPA, Grade Point Average, grades were recoded so that higher GPA reflects better academic achievement. Correlations of the residuals: r1 × r2: r = 0.25; r1 × r3: r = 0.06; r2 × r3: r = 0.03. ^∗^p ≤ 0.01 and ^∗∗^*p* ≤ 0.001*.

In line with Hypothesis 2, school climate predicted both SWB component above and beyond self-efficacy, interest, and test anxiety. Here, general school climate yielded the highest effect of all exogenous variables on both SWB factors. Furthermore, mood was still significantly associated with self-efficacy after all other variables were controlled for. Life satisfaction was additionally significantly associated with worry. When all other variables were controlled for, GPA was only significantly associated with self-efficacy and only marginally with school climate and test anxiety but none of the other specific school climate variables or interest were.

## Discussion

The aim of the present paper was to shed further light on the importance of school-related and individual factors for two important school outcomes (i.e., academic achievement and SWB). To this end, we investigated the bivariate and incremental criterion validity of school climate, academic self-efficacy, interest, and test anxiety in explaining interindividual differences in academic achievement and SWB.

### The Construct and Criterion Validity of School Climate

Research Questions 1a and 1b addressed the construct validity of school climate and its association with SWB and academic achievement. With a nested factor school climate model, we demonstrated that specific school climate factors did not incrementally contribute to explaining variance in SWB or in academic achievement. The general school factor was the only predictor of both criteria. Consequently, even when school climate is assessed with different subscales, future studies should use a global school climate factor rather than different school climate subscales because the subscales are highly correlated (e.g., Salmela-Aro et al., 2008). When using highly correlated predictors, the problem of multicollinearity might occur, and this makes it difficult to interpret the different paths to a criterion or criteria (see Marsh et al., 2004). Furthermore, when modeling specific but correlated factors, one does not know whether the explained variance can be attributed to this specific school factor or to a higher order factor that can explain both the correlations between the specific factors and the correlations with the criterion or criteria (see Brunner et al., 2012). Nested factor models (e.g., Brunner et al., 2012) solve this problem. Thus, we strongly encourage other researchers to also use nested factor models when investigating school climate.

The bivariate correlations demonstrated that general school climate was associated with GPA and both SWB components. Several theories for explaining SWB refer to the relevance of the environment (e.g., family, peers, school; see Bronfenbrenner, 1979; Baker et al., 2003). However, regarding academic achievement, there is a lot of empirical research demonstrating that individual characteristics such as students’ motivation play a particularly important role (Steinmayr et al., 2014). Hence, in Hypothesis 1, we supposed that the school environment (i.e., the school climate) would be a stronger predictor of different components of SWB than of academic achievement. This hypothesis was only partly confirmed. Our analyses showed that just the path coefficient from general school climate to mood was significantly higher than the coefficients from general school climate to academic achievement and from general school climate to life satisfaction. Even though Hypothesis 1 was only partly corroborated, the results still extend our knowledge on how school climate is associated with SWB and academic achievement. Importantly, this is one of the first studies to demonstrate the relevance of school climate for SWB by considering not only affective but also cognitive SWB components. Previous studies on school climate have focused on variables that are strongly related to SWB such as school burnout (e.g., Salmela-Aro et al., 2008) rather than on SWB itself. Hence, our study extends previous findings and demonstrates the relevance of assessing affective as well as cognitive components of SWB because they have differential associations with their various determinants (Diener, 2012), among them school climate (see Aldridge et al., 2016). In this context, our results support the theoretical assumptions about the relevance of the environment for children’s and adolescents’ well-being (Eccles et al., 1993). In this context, our findings tentatively suggest that positive surroundings including school climate might be more relevant for enhancing happiness and positive feelings (the affective component of SWB) than for enhancing life satisfaction or academic achievement. Regarding life satisfaction, research has demonstrated that personality and other individual variables seem to be especially relevant (see Lucas, 2008), whereas, for example, family variables, as another aspect of the environment, play a more important role in affective variables of well-being (Lucas, 2008). Thus, school climate might indeed be more important for the affective component of well-being than for the cognitive component as our results suggest. In addition, the fact that the path from school climate to life satisfaction was not statistically different from the path from school climate to GPA might also be explained by methodological issues. As reported above, the highest percentage of missing values occurred for GPA. Thus, among all variables, most of the missing values had to be estimated here. Estimating missing values for a variable leads to an increase in the standard error, which makes it harder to find significant associations between two variables or to find differences in path weights. The path from school climate to life satisfaction and the path from school climate to academic achievement may have been significantly different if less data had been missing for GPA. Summing up, the present study demonstrated the importance of general school climate for both components of SWB and academic achievement even though it importance varies between criteria. Given that a central goal of our society is to further enhance children’s and adolescents’ SWB beside academic achievement, our study shows a promising approach for reaching this goal by focusing on a positive school climate (see Suldo et al., 2013).

### The Incremental Validity of School Climate

Whether school climate explains more variance in SWB and academic achievement than individual characteristics do has been an open question. In the present study, we focused on student characteristics (e.g., self-efficacy, interest, and test anxiety) as important for both SWB and academic achievement (e.g., Pekrun et al., 2017). No previous study has investigated school climate variables as well as important student characteristics simultaneously with regard to different components of SWB and academic achievement. On the basis of Suldo et al. (2008) assumptions, we expected school climate to predict SWB above and beyond self-efficacy, interest, and test-anxiety (Hypothesis 2). In line with this assumption, general school climate was the strongest predictor of both of the SWB components above and beyond self-efficacy, interest, and test anxiety. Mood was still significantly associated with self-efficacy after all other variables were controlled for. Regarding life satisfaction, just self-efficacy showed effects after we controlled for the other student characteristics and school climate as the most relevant factor. These results point to the fact that SWB is a multicausal phenomenon, and no single variable can explain whether a person is happy or satisfied (see Eid and Larsen, 2008).

Furthermore, on the basis of the opportunity-propensity framework by Byrnes and Miller (2007), we hypothesized that individual student characteristics important for school success such as self-efficacy, interest, and test anxiety would predict academic achievement above and beyond school climate (Hypothesis 3). This part of the hypothesis was only partly confirmed: Although self-efficacy and test anxiety components (marginally significantly) explained variance in academic achievement above and beyond school climate, interest did not. Thus, our results especially confirm the relevance of self-efficacy beliefs for academic achievement in relation to other motivational variables and school climate (Pajares, 2006). The fact that interest did not longer yield an effect on GPA after all other variables were controlled for might partly be attributable to its high correlations with self-efficacy (see also Steinmayr et al., 2018). In case of multicollinearity it might be that the importance of predictors are underestimated as both predictors explain a share of variance in the criterion together (cf. Steinmayr and Spinath, 2009). However, school climate was also correlated with self-efficacy (see also Henry et al., 2011) but, as expected, did not predict academic achievement after all other variables were controlled for (see also Alivernini and Lucidi, 2011; Wang et al., 2013). Thus, it might be that fostering, for example, students’ school climate might also have a positive impact on other variables related to academic achievement such as self-efficacy (see Henry et al., 2011 for results pointing in that direction). Given that longitudinal research has shown that school climate is a determinant of academic achievement (see McCoy et al., 2013), when other variables are not controlled for, interventions enhancing a positive climate at school might not only enhance students’ SWB but also, indirectly via factors such as self-efficacy, their academic achievement. Longitudinal studies show an impact on school climate factors such as student-teacher relationship on students’ self-efficacy (Alivernini and Lucidi, 2011) which further underlines this thought.

### Limitations and Directions for Future Research

Although the findings are promising, our study also has several limitations. First, the study was cross-sectional. Thus, no causal conclusions can be drawn from the present study. Nonetheless, the study provides valuable insights into the relations of school climate, self-efficacy, interest, test anxiety, SWB, and academic achievement, constructs that hitherto have not been investigated in concert. Showing significant correlations is the first step in investigating their relations because a correlation is a necessary albeit not a sufficient precondition for demonstrating a causal relationship.

Second, we measured school climate with only one questionnaire. Thus, the specification of school climate as a nested factor model has thus far been shown only for this particular measure. Even though it is a well-established and validated German measure (Eder, 1998), future studies should determine whether a nested factor model also fits the data equally well when other school climate questionnaires are used. This would also open up the opportunity to investigate the importance of other specific school climate factors, for example, student–student relationships for predicting SWB and academic achievement beyond a general school climate factor.

Third, we demonstrated that school climate predicted academic achievement beyond other student characteristics that are relevant for academic achievement. Here, we chose only constructs that had the potential to be related to both SWB and academic achievement. We did not include broader personality factors such as the Big Five, which are powerful predictors of SWB (e.g., Soto, 2015) because the personality trait that has the strongest association with SWB (i.e., neuroticism) is not related to academic achievement (e.g., Poropat, 2009). Moreover, the only personality trait that was previously found to be associated with grades in the age range comparable to the age range in our sample (i.e., conscientiousness) does not appear to be related to SWB (Gomez et al., 2009). Besides not considering the Big Five, we excluded student characteristics that are important for academic achievement (e.g., intelligence) because such characteristics have not be found to be associated with SWB (for intelligence, see Fischbach et al., 2013). However, future studies that concentrate on either SWB or academic achievement should investigate whether school climate also incrementally contributes to the prediction of academic achievement and SWB when additional student characteristics that were not considered in the present study (e.g., personality traits) are included. Furthermore, we did not consider constructs that are conceptually close to the investigated constructs that are related to both grades and SWB (e.g., self-concept or expectations of success). Given that a recent study demonstrated that expectations of success explained more variance in GPA than self-efficacy did (Steinmayr et al., 2018), it might be the case that results would turn out differently, at least concerning the prediction of GPA, if constructs other than the ones chosen in the present study were considered.

Fourth, we demonstrated the importance of school climate and the other variables for academic achievement operationalized as GPA. However, if other indicators of academic achievement such as standardized achievement tests are considered, the results might change because both student characteristics and school-related factors show differential associations with different indicators of school achievement (Steinmayr et al., 2014).

Last but not least the sample was only recruited from four schools. This had at least two implications. First, data were nested in schools (not for all students in classes as described above) but due to the low amount of schools we could not control for this data structure in the analysis. Even though we controlled for potential school effects by z-standardizing, due to the low number of clusters we did not apply a statistical procedure properly taking into account non-independence of the investigated schools samples, which might have affected standard errors. Thus, future studies should investigate larger cohorts covering more schools to deal with that problem. Furthermore, the sample we investigated was representative for the schools we investigated but the investigated student population was not representative for all students of the age range investigated. Students in the present study were from families with higher educational background and more rarely had a migration background. These background variables are known for influencing academic achievement (for example Steinmayr et al., 2010) whereas studies are inconsistent regarding their relationship to SWB (for an overview, see Crede et al., 2015). Thus it might be that results in a representative sample would differ from those reported here, which should be addressed in future studies.

## Conclusion

Summing up, this cross-sectional study offers valuable insights into the interplay of school-related and individual characteristics with regard to SWB and academic achievement. SEM revealed that a positive school climate, self-efficacy, and the worry component of test anxiety predicted SWB and/or GPA after other important variables were controlled for. Thus, our findings suggest that it is not enough to concentrate on only environmental aspects (e.g., school climate) or student characteristics when explaining interindividual differences in adolescents’ SWB and academic achievement. Instead, both criteria are related to both school-related and individual characteristics and should be taken into account in future studies. The associations revealed in this cross-sectional study further form the basis for future research testing for causal relations among the constructs and developing potential approaches to foster students’ healthy academic development and their personal development simultaneously. Our findings thereby speak to the relevance of creating a positive school climate (see Baker et al., 2003; Cohen et al., 2009) for enhancing well-being and, indirectly for example via self-efficacy, educational attainments such as GPA.

## Data Availability Statement

The raw data supporting the conclusions of this manuscript will be made available by the authors, without undue reservation, to any qualified researcher.

## Ethics Statement

This study was carried out in accordance with the recommendations of the German Research Foundation. The protocol was not approved by an ethic committee as this was not required. However, we followed all guidelines given by the local ethic committee of the TU Dortmund regarding studies with human-beings, especially children. All parents of subjects gave written informed consent in accordance with the Declaration of Helsinki.

## Author Contributions

RS had the idea and provided a first draft of the manuscript and finalized the paper. AH wrote the section on individual characteristics predicting academic achievement and gave valuable suggestions for improving the first draft. JM wrote part of the method section and read and commented the first draft. CN set up the study together with RS, went to schools and tested, data management, read and commented the first draft. LW wrote the section on individual characteristics predicting subjective well-being and discussed Hypotheses 3 and 4, gave valuable suggestions for improving the first draft.

## Conflict of Interest Statement

The authors declare that the research was conducted in the absence of any commercial or financial relationships that could be construed as a potential conflict of interest.
